# Sleep patterns, genetic susceptibility, and venous thromboembolism: A prospective study of 384,758 UK Biobank participants

**DOI:** 10.1371/journal.pone.0309870

**Published:** 2024-09-06

**Authors:** Jiaxin Bai, Ziyu Yang, Yu Jia, Jing Yu, Wenli Jiang, Yi Liu, Fanghui Li, Rui Zeng, Zhi Wan, Yi Lei, Xiaoyang Liao, Dongze Li, Qian Zhao

**Affiliations:** 1 General Practice Ward/International Medical Center Ward, General Practice Medical Center, West China Hospital, Sichuan University, Chengdu, China; 2 Department of Emergency Medicine, West China Hospital, Sichuan University, Chengdu, Sichuan, China; 3 Department of Cardiology, West China Hospital, West China School of Medicine, Sichuan University, Chengdu, Sichuan, China; RWJBH: RWJBarnabas Health, UNITED STATES OF AMERICA

## Abstract

**Background:**

Although healthy sleep patterns have been linked to a lower risk of cardiovascular disease in earlier research, it is unclear how beneficial they are for venous thromboembolism (VTE).

**Aim:**

This research aimed to examine the correlation between sleep patterns, genetic susceptibility, and VTE.

**Methods:**

In the UK Biobank cohort, healthy sleep behaviors were defined as early chronotype, 7–8 hours of sleep each day, no snoring, infrequent insomnia, and infrequent daytime sleepiness. Each of the five criteria was given 1 point, creating a healthy sleep score ranging from 0 to 5. Cox proportional hazards regression models were utilized to examine the associations between genetic susceptibility, healthy sleep score and VTE.

**Results:**

The UK Biobank study included 384,758 participants aged 56.6 ± 8.0 years. After a median of 11.9 years of follow-up, 8,885 (2.3%) participants were diagnosed with VTE. A healthy sleep score inversely affected VTE risk. For participants with a score of 5, the hazard ratio of VTE was 0.813 (95% confidence interval: 0.758–0.873, P<0.001) compared to those with a score ≤2. Early chronotype, sleeping 7–8 hours each day, infrequent insomnia, and infrequent daytime sleepiness were significantly associated with a 7.9%, 8.3%, 5.1%, and 20.7% lower risk of VTE, respectively. In addition, the correlation between sleep pattern and the incidence of VTE was consistent, regardless of genetic susceptibility (P for interaction = 0.366).

**Conclusions:**

Our secondary analysis of a large-scale prospectively gathered registry revealed that individuals with a healthy sleep pattern are significantly correlated with lower risk of developing VTE, irrespective of genetic susceptibility.

## Introduction

With more than 10 million cases per year worldwide, venous thromboembolism (VTE), including pulmonary embolism (PE) and deep vein thrombosis (DVT), ranks as the third most prevalent cardiovascular ailment [1]. A large lower extremity vein might develop single or multiple thromboses, which is known as DVT [2]. PE is the most dangerous VTE that occurs when an embolus enters the lungs from a vein and occludes the pulmonary artery [3]. Approximately 20% of individuals with a VTE die within 1 year, and various complications are common among survivors [4]. Therefore, enhancing the prediction and prevention of VTE by identifying its risk factors is critical.

Hospital-based risk factors (e.g., congestive heart failure, recent prior surgery and cancer) for VTE have recently been identified, and effective preventive measures, both primary and secondary, have been given to high-risk patients; however, VTE seems to occur constantly or even increase [1]. The prevention of VTE has expanded beyond targeting primarily hospital-based risk factors to avoid harmful lifestyles, such as smoking and obesity [5]. Adequate evidence has proven that several unhealthy sleep behaviors [6], including long or short sleep duration [7, 8], late chronotype [9, 10], insomnia [11–13], snoring [14], and frequent daytime sleepiness [15], are significant risk factors for cardiovascular disease, primarily coronary heart disease, stroke, and myocardial infarction [16]. However, the etiology of VTE is distinct from that of arterial thrombosis, and there is a paucity of research on the protective effects of healthy sleep habits against VTE.

It is generally accepted that behavioral and genetic factors play a role in cardiovascular disease, including VTE [17]. Numerous studies have demonstrated that lifestyle factors may interact with genetic susceptibility to cardiovascular disease. Furthermore, healthy lifestyle choices such as reducing alcohol consumption [18], quitting smoking [19], engaging in appropriate physical activity [20], and avoiding obesity [21] may significantly reduce the genetically predetermined risk of VTE.

However, whether healthy sleep patterns, which incorporate a variety of sleep behaviors, such as early chronotype, 7–8 hours of sleep each day, infrequent insomnia, no snoring, and infrequent daytime sleeping, could mitigate the impact of genetic susceptibility on VTE remains unknown. Thus, using the UK Biobank cohort project, we prospectively explored the relationships between particular main sleep behaviors, as measured by a healthy sleep score, and VTE risk. Furthermore, our objective was to investigate the possible correlation between genetic susceptibility and healthy sleep patterns, as well as their combined affiliation with VTE (PE and DVT).

## Materials and methods

### Study population

The data utilized in this study were acquired from the UK Biobank, which is a large-scale prospective cohort study with a sufficiently representative sample of the UK population. A total of 502,655 individuals aged 37 to 73 years were included in the UK Biobank between 2006 and 2010. The participants were recruited from 22 assessment sites situated throughout England, Wales and Scotland. These individuals self-completed a touchscreen questionnaire and participated in verbal interviews to provide information on their sociodemographics and lifestyle. They also completed a number of physiological and anthropometric measurements [22]. Specifics on the acquisition of data may be found at the following website: http://www.ukBiobank.ac.uk. Materials and data are available at https://ukBiobank.dnanexus.com/panx/projects..

A total of 242 people chose to discontinue their participation in the UK Biobank project. In this analysis, we removed individuals who were not white (n = 23,365; addressing the influence of genetic variations among different racial groups on the research outcomes), those who had a history of prevalent VTE at the onset of the study (n = 3,940), and those who had missing data to calculate sleep (n = 84,460) and polygenic risk scores (n = 5,890). A total of 384,758 participants were ultimately included in our final prospective analysis.

### Ethics statement

The UK Biobank was ethically approved for this study by the NHS National Research Ethics Service (16/NW/0274). The experimental protocols were carried out in accordance with the ethical guidelines of the Helsinki Declaration. Written informed consent was obtained from the participants or their guardians. All methods were established under guidelines and regulations developed by the UK Biobank. The use of the data was approved by the Human Ethical Committee of the West China Hospital of Sichuan University (2021–601).

### Patient and public involvement

The participants in this study were not engaged in the process of designing, implementing, reporting, or disseminating the research.

### Assessment of sleep behaviors

During recruitment, a touchscreen questionnaire was utilized to gather all self-reported information on sleep behaviors, including sleep chronotype, sleep duration, snoring, insomnia, and frequent daytime sleeping.

The evaluation of chronotype preference was carried out by asking "How do you perceive yourself?" Participants were asked to categorize themselves as follows: "certainly a ’morning’ person," "morning-oriented more than evening-oriented," "evening-oriented more than morning-oriented," or "certainly an ’evening’ person." Furthermore, each participant’s sleep duration was assessed by the exact number of hours they slept over the course of 24 hours, naps included. The symptoms of insomnia were determined through an inquiry into whether the individual had difficulty initiating sleep or awakening during the night. The response options for the questions were "usually," "sometimes," or "never/rarely." The data pertaining to snoring were acquired through an inquiry into whether the participant’s close relative, companion, or acquaintance lodges complaints regarding their snoring. Participants were given the option to answer either "yes" or "no." Subsequently, "To what extent do you believe it is likely that you will inadvertently fall asleep or doze off during the day?" was utilized to collect information pertaining to participants’ daytime sleepiness. The options for replies were categorized as “Never/rarely,” “Sometimes,” "Often, and “All of the time.” In the event that the sleep habits indicated above underwent significant changes, participants were requested to provide an average response to their behavior in the last month.

### Definition of a healthy sleep pattern and healthy sleep score

The criteria for healthy sleep pattern were established as having an early chronotype (being more of a "morning" person than an "evening" person), getting 7–8 hours of sleep each day, experiencing infrequent insomnia ("sometimes" or "never/rarely"), not snoring, and experiencing infrequent daytime sleepiness ("sometimes" or "never/rarely"). The participants received 1 point for each instance of healthy sleep behavior, provided that they matched the specified requirements. Otherwise, they received 0 points. The healthy sleep score, which ranges from 0 to 5, is computed by adding together the individual scores of each of the five components. A higher score indicates a healthier sleep pattern [23].

### Assessment of VTE

Participants who had the occurrence of DVT or PE during follow-up were categorized as having developed VTE in this study. The results were chiefly identified by analyzing hospital inpatient records, which included information on admissions and diagnosis. These records were obtained from the Hospital Episode Statistics for the United Kingdom, the Patients Episode Database for Wale, and Scottish Morbidity Record data for Scotland. According to the International Classification of Diseases Edition 10, we identified the following outcomes: I26 for PE and I80, I81, and I82 for DVT.

Following enrollment, the follow-up time was calculated from that date until the earliest of the following occurred: first diagnosis of VTE, death, or termination of the current follow-up in 2023.

### Definition of the polygenic risk score

The Polygenic Risk Score (PRS) is a numerical assessment of an individual’s genetic susceptibility to a certain disease. Comprehensive details about quality control, genotyping, and imputation in the UK Biobank research have already been documented [24]. To compute the VTE PRS, a weighted method was implemented. In this investigation, we developed a PRS for VTE by utilizing 35 specific genetic variations called single nucleotide polymorphisms (SNPs), which were derived from a prior genome-wide association study [25]. Afterwards, the individuals’ SNPs were transformed into numerical values of 0, 1, or 2 concerning the count of risk alleles. The impact of SNPs (β) on VTE was utilized as the metric to calculate the aggregated sum of all selected SNPs for the PRS. The VTE PRS equation was: PRS = (β1 *SNP1 + β2 *SNP2 + … + β35 *SNP35). The VTE PRS ranged from −3.98 to 6.23. Based on the PRS tertile, participants were split into three subgroups: low-risk, intermediate-risk, and high-risk.

### Assessment of covariates

Drawing on the literature, we identified a number of sociodemographic and behavioral confounders. A variety of health-related data, such as sex, age, education level, income, physical activity, body mass index, drinking and smoking status, cardiovascular disease, hypertension, diabetes, cancer, blood glucose levels, triglyceride levels, total cholesterol, low-density lipoprotein cholesterol, and high-density lipoprotein cholesterol, were collected from the UK Biobank.

Based on height and weight data, the body mass index was computed. A systolic/diastolic blood pressure ≥140/90 mmHg was regarded as hypertension, as was the use of antihypertensive medications. A blood glucose level of 126 mg/dL during fasting, 200 mg/dL during nonfasting, or hypoglycemic medication use was considered to indicate diabetes. Cardiovascular disease markers in this research included self-reported past coronary procedures, anomalies on physiological tests or electrocardiograms, and confirmed incidences of cardiovascular disease.

### Statistical analysis

Both parametric and non-parametric continuous variables were presented as means ± standard deviations or medians (25th, 75th), respectively, and were compared utilizing analysis of variance or the Mann‒Whitney U test. Categorical variables were reported as n (%) and compared utilizing the chi-squared test. The missing continuous variables were filled in utilizing multivariate imputation using a chained equation employing predictive mean matching [26].

The minimum sample size was calculated by using the log-rank test [27]. When the type I error and type II error are 0.05 and 0.20, respectively, the calculated minimum sample size required was 35,376.

Individuals were grouped into 4 groups based on their healthy sleep scores: 0–2, 3, 4, or 5. However, individuals who scored 0 and 1 were not suitable for independent analysis due to their low representation in the entire population (0.2% and 2.2%, respectively). We used Cox proportional hazards models to derive hazard ratios (HRs) and 95% confidence intervals (CIs) to evaluate the correlation between healthy sleep patterns and VTE. The reference group consisted of individuals with scores ranging from 0 to 2. Furthermore, to address the possibility of non-vascular disease mortality preventing the incidence of VTE when it occurs first, we used the Gray-fine subdistribution hazard model to evaluate the relationship between healthy sleep score and VTE. For the purpose of investigating the correlation between individual sleep habits and VTE, we included all five components in the model concurrently, treating each one as a binary variable—either meeting or not meeting the healthy criteria. Similar analyses were repeated for participants diagnosed with PE or DVT. All models were fully adjusted for sex, age, income, education, physical activity, body mass index, drinking and smoking status, hypertension status, diabetes status, cancer status, cardiovascular disease status, blood glucose levels, triglyceride levels, low-density lipoprotein cholesterol, high-density lipoprotein cholesterol and total cholesterol. Sensitivity analyses were conducted among individuals without missing values and those who were followed up for more than 2 years. Moreover, cumulative incidence functions were used to estimate the cumulative probability of VTE (PE or DVT) events in four groups with different healthy sleep scores. Referring to previous research, two-sided Gray’s test was utilized to compare the results among the four groups [28].

A fully adjusted Cox proportional hazard model was conducted to examine the correlation between an individual’s healthy sleep score and their risk of developing VTE among different genetic risk subgroups. Additionally, an interaction test was performed to determine whether there was any interaction effect between an individual’s healthy sleep score and their genetic susceptibility to VTE. Furthermore, we evaluated the combined impact of the PRS and healthy sleep score on the risk of VTE utilizing a Cox proportional hazard model.

SPSS version 26.0 (IBM Corp, Armonk, NY, USA) and R statistical software (version 4.1.1) were applied for all the statistical analyses. For all tests, a two-tailed P value less than 0.05 was considered to indicate statistical significance.

## Results

### Patient characteristics

This study included 384,758 participants aged 56.6 ± 8.0 years, 172598 (44.9%) of whom were male. After an average of 11.9 years of follow-up, 8,885 (2.3%) patients were diagnosed with VTE, with 5,103 (1.3%) and 4,567 (1.2%) patients diagnosed with PE and DVT, respectively. The baseline parameters were compared based on the occurrence of VTE (Table 1). Participants with VTE exhibited characteristics such as older age, lower education level, higher proportion of males, socioeconomically disadvantaged status, higher prevalence of smoking, lower likelihood of engaging in physical activity, and lower prevalence of current alcohol use compared to those without VTE (P<0.05). Furthermore, they exhibited an increased incidence of cardiovascular risk factors, including hypertension, diabetes, cardiovascular disease, and cancer, at the outset of the study (P<0.05).

**Table 1:** Baseline population characteristics in the UK Biobank study (2006–2010).

Characteristic	Non -VTE	VTE	P
	(N = 375873)	(N = 8885)	
**Demographic Variables**			
Age, years	56.5 (8.05)	60.0 (7.09)	<0.001
Male sex	167975 (44.7)	4623 (52.0)	<0.001
MET hour all	40.73 (9.9–57.58)	40.04 (7.85–57.41)	0.144
Education			<0.001
College or University degree	121260 (31.5)	2199 (24.7)	
A levels/AS levels or equivalent	42447 (11.3)	814 (9.2)	
O levels/GCSEs or equivalent	102644 (27.3)	2342 (26.4)	
Other (e.g.NVO,nursing, missing)	109522 (29.1)	3530 (39.7)	
Income, US			<0.001
<£18 000	88437 (23.5)	2891 (32.5)	
£18 000 to £52 000	190420 (50.7)	4530 (51.0)	
>£52 000	97016 (25.8)	1464 (16.5)	
Drinking			<0.001
Never	11987 (3.2)	374 (4.2)	
Former	12544 (3.3)	397 (4.5)	
Current	351342 (93.5)	8114 (91.3)	
Smoking			<0.001
Never	202710 (53.9)	4246 (47.8)	
Former	134157 (35.7)	3547 (39.9)	
Current	39006 (10.4)	1092 (12.3)	
**Chronic Medical Conditions**			
Hypertension	98580 (26.2)	3064 (34.5)	<0.001
Diabetes mellitus	17340 (4.6)	629 (7.1)	<0.001
Cancer	31155 (8.3)	1173 (13.2)	<0.001
Cardiovascular disease	105494 (28.1)	3294 (37.1)	<0.001
**Physiological and Lab Variables**			
Body mass index, kg/m^2^	27.2 ± 5.20	28.4 ± 5.98	<0.001
Systolic blood pressure, mmHg	135.1 ± 26.61	136.5 ± 28.45	<0.001
Diastolic blood pressure, mmHg	80.6 ± 15.14	80.9 ± 16.28	0.055
Total cholesterol, mmol/l	5.62 ± 1.31	5.52 ± 1.38	<0.001
high density lipoprotein cholesterol, mmol/l	1.44 ± 0.39	1.38 ± 0.38	<0.001
low density lipoprotein cholesterol, mmol/l	3.52 ± 0.93	3.46 ± 0.97	<0.001
Triglycerides, mmol/l	1.72 ± 1.02	1.83 ± 1.02	<0.001
Blood glucose, mmol/l	4.90 ± 1.47	4.96 ± 1.69	0.002

Values are expressed as n (%), mean (SD), and median (25^th^-75^th^).

### Healthy sleep score, VTE, and non-vascular disease mortality

**Fig 1:**
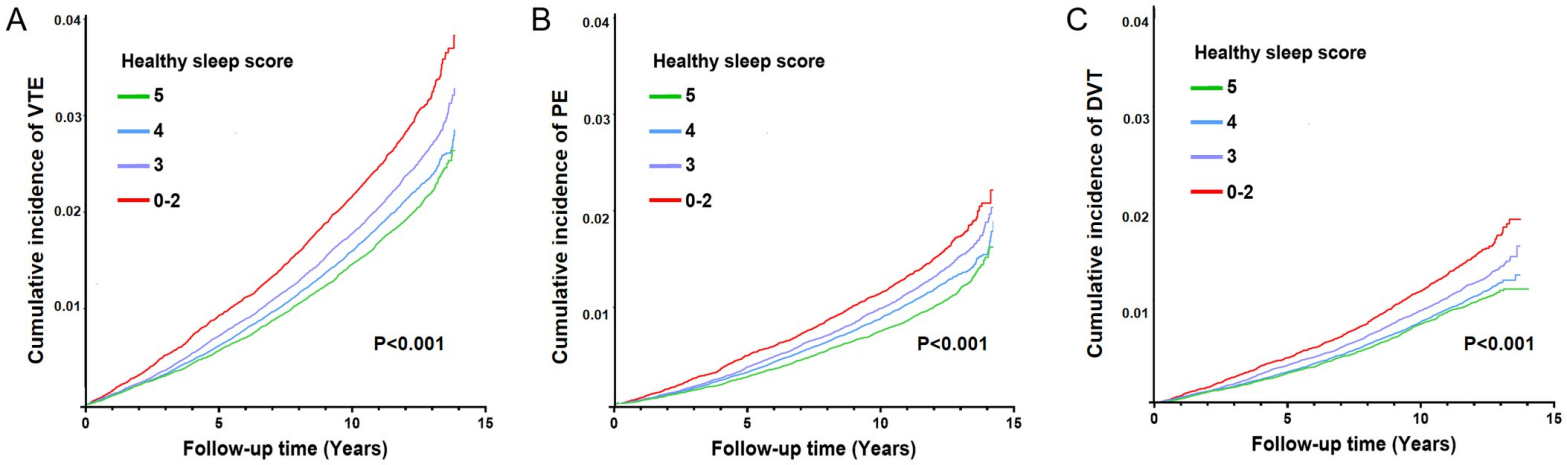
The cumulative incidence of VTE (PE and DVT) when addressing he non-vascular disease mortality in different healthy sleep score groups. A. Cumulative incidence of VTE with different healthy sleep scores; B. Cumulative incidence of PE with different healthy sleep scores; C. Cumulative incidence of DVT with different healthy sleep scores.

After eliminating the competitive risk of non-vascular disease mortality, Fig 1A shows that the cumulative incidence of VTE varied significantly across different healthy sleep score groups (P < 0.001, Fig 1), indicating a progressive increase in VTE incidence as the healthy sleep score decreased. Comparable patterns were also evident in the cumulative incidence of PE and DVT (Fig 1).

The results of the Cox proportional hazards regression analysis are shown in Table 2. According to the fully adjusted Model 3, there was a negative correlation between a healthy sleep score and VTE (HR for 1-point rise: 0.935, 95% CI: 0.917–0.955, P<0.001). Among those with a healthy sleep score of 5, the HR of VTE decreased to 0.813 (95% CI: 0.758–0.873, P<0.001), compared with that of the reference group (scores of 0–2). When assessing the correlation between each binary (healthy vs. unhealthy) factor of the healthy sleep pattern and the risk of VTE, the HRs of VTE were 0.921 (95% CI: 0.881–0.964, P<0.001) for 7–8 hours of sleep each day, 0.917 (95% CI: 0.878–0.957, P<0.001) for early chronotype, 0.949 (95% CI: 0.906–0.994, P = 0.028) for infrequent insomnia, and 0.793 (95% CI: 0.712–0.882, P<0.001) for infrequent daytime sleepiness compared with their counterparts. However, no snoring was not significantly associated with VTE (HR: 0.996, 95% CI: 0.953–1.041, P = 0.853) (Table 2).

**Table 2:** Hazard ratios of VTE by healthy sleep score and its individual components among 384758 UK Biobank participants.

Sleep behaviors	No. of events/total No. (n, %)	Model 1		Model 2		Model 3	
		HR (95% CI)	P	HR (95% CI)	P	HR (95% CI)	P
**Healthy sleep score**			<0.001		<0.001		<0.001
0–2	1530/52500 (2.9%)	Reference	-	Reference	-	Reference	-
3	2632/108180 (2.4%)	0.827 (0.776–0.880)	<0.001	0.836 (0.785–0.890)	<0.001	0.877 (0.823–0.934)	<0.001
4	3075/141555 (2.2%)	0.734 (0.691–0.781)	<0.001	0.765 (0.719–0.813)	<0.001	0.826 (0.776–0.879)	<0.001
5	1648/82523 (2.0%)	0.672 (0.627–0.720)	<0.001	0.727 (0.678–0.780)	<0.001	0.813 (0.758–0.873)	<0.001
Per 1 point		0.882 (0.864–0.899)	<0.001	0.904 (0.886–0.922)	<0.001	0.935 (0.917–0.955)	<0.001
**Individual component** *							
Chronotype							
Late chronotype	3379/143370 (2.4%)	Reference		Reference		Reference	
Early chronotype	5506/241388 (2.3%)	0.970 (0.926–1.013)	0.168	0.894 (0.857–0.934)	**<0.001**	0.917 (0.878–0.957)	**<0.001**
Sleep duration							
<7h/d or 8h/d<	3166/121259 (2.6%)	Reference		Reference		Reference	
7–8 h/d	5719/263499 (2.2%)	0.852 (0.814–0.891)	**<0.001**	0.895 (0.856–0.936)	**<0.001**	0.921 (0.881–0.964)	**<0.001**
Frequent insomnia							
Yes	2775/107723 (2.6%)	Reference		Reference		Reference	
No	6110/277035 (2.2%)	0.891 (0.851–0.934)	**<0.001**	0.927 (0.884–0.971)	**0.001**	0.949 (0.906–0.994)	**0.028**
Snoring							
Yes	3591/142768 (2.5%)	Reference		Reference		Reference	
No	5294/241990 (2.2%)	0.873 (0.837–0.911)	**<0.001**	0.936 (0.896–0.978)	**0.003**	0.996 (0.953–1.041)	0.853
Frequent daytime sleepiness							
Yes	357/10116 (3.5%)	Reference		Reference		Reference	
No	8528/374641 (2.3%)	0.675 (0.607–0.751)	**<0.001**	0.753 (0.677–0.838)	**<0.001**	0.793 (0.712–0.882)	**<0.001**

Model 1 is univariable Cox proportional hazards regression analysis.

Model 2 is adjusted by age (continuous, years), sex (male, female), education (College or University degree, A levels/AS levels or equivalent, O levels/GCSEs or equivalent, Other (e.g., NVO, nursing, missing)), and annual household income (<£18 000, £18 000 to £52 000, >£52 000).

Model 3 is adjusted by Model 2 plus body mass index (continuous, kg/m2), physical activity (continuous, MET-hours/week), smoking (never, former, current), drinking (never, former, current), hypertension (y/n), diabetes (y/n), cancer (y/n), cardiovascular disease (y/n), total cholesterol (continuous, mmol/l), high-density lipoprotein cholesterol (continuous, mmol/l), low-density lipoprotein cholesterol (continuous, mmol/l), triglycerides (continuous, mmol/l) and blood glucose (continuous, mmol/l). HR, hazard ratio; CI, confidence interval; Ref, reference; y/n, yes/no; VTE, venous thromboembolism.

*Each individual component was modeled as binary variable: met or unmet the healthy criterion. All the five individual components were included in the model simultaneously.

When using Gray-fine subdistribution hazard model to eliminate the non-vascular disease mortality in the competing risk analysis (Table 3), the results showed a significant negative correlation between the healthy sleep score and VTE (HR for 1-point rise: 0.939, 95% CI: 0.919–0.960, P<0.001). Moreover, the relationships between sleep-related variables and VTE were largely consistent.

**Table 3:** Hazard ratios of VTE by healthy sleep score and its individual components among 384758 UK Biobank participants (non-vascular disease mortality was addressed).

Sleep behaviors	No. of events/total No. (n, %)	Model 1		Model 2		Model 3	
		HR (95% CI)	P	HR (95% CI)	P	HR (95% CI)	P
**Healthy sleep score**			<0.001		<0.001		<0.001
0–2	1530/52500 (2.9%)	Reference	-	Reference	-	Reference	-
3	2632/108180 (2.4%)	0.879 (0.859–0.899)	<0.001	0.902 (0.881–0.923)	<0.001	0.939 (0.916–0.962)	<0.001
4	3075/141555 (2.2%)	0.860 (0.834–0.887)	<0.001	0.879 (0.852–0.906)	<0.001	0.915 (0.886–0.944)	<0.001
5	1648/82523 (2.0%)	0.830 (0.780–0.884)	<0.001	0.842 (0.791–0.897)	<0.001	0.885 (0.830–0.943)	<0.001
Per 1 point		0.881 (0.862–0.900)	<0.001	0.905 (0.885–0.925)	<0.001	0.939 (0.919–0.960)	<0.001
**Individual component** *							
Chronotype							
Late chronotype	3379/143370 (2.4%)	Reference		Reference		Reference	
Early chronotype	5506/241388 (2.3%)	0.971 (0.931–1.014)	0.180	0.899 (0.861–0.939)	**<0.001**	0.919 (0.880–0.960)	**<0.001**
Sleep duration							
<7h/d or 8h/d<	3166/121259 (2.6%)	Reference		Reference		Reference	
7–8 h/d	5719/263499 (2.2%)	0.857 (0.820–0.896)	**<0.001**	0.901 (0.862–0.943)	**<0.001**	0.928 (0.888–0.971)	**0.001**
Frequent insomnia							
Yes	2775/107723 (2.6%)	Reference		Reference		Reference	
No	6110/277035 (2.2%)	0.894 (0.854–0.937)	**<0.001**	0.930 (0.888–0.974)	**0.002**	0.956 (0.912–1.001)	0.055
Snoring							
Yes	3591/142768 (2.5%)	Reference		Reference		Reference	
No	5294/241990 (2.2%)	0.872 (0.836–0.910)	**<0.001**	0.929 (0.890–0.971)	**<0.001**	0.991 (0.949–1.036)	0.700
Frequent daytime sleepiness							
Yes	357/10116 (3.5%)	Reference		Reference		Reference	
No	8528/374641 (2.3%)	0.684 (0.615–0.761)	**<0.001**	0.763 (0.685–0.849)	**<0.001**	0.805 (0.723–0.896)	**<0.001**

Model 1 is univariable Competing risk analysis.

Model 2 is adjusted by age (continuous, years), sex (male, female), education (College or University degree, A levels/AS levels or equivalent, O levels/GCSEs or equivalent, Other (e.g.NVO,nursing,missing)), annual household income (<£18 000, £18 000 to £52 000, >£52 000).

Model 3 is adjusted by Model 2 plus body mass index (continuous, kg/m2), physical activity (continuous, MET-hours/week), smoking (never, former, current), drinking (never, former, current), hypertension (y/n), diabetes (y/n), cancer (y/n), cardiovascular disease (y/n), total cholesterol (continuous, mmol/l), high-density lipoprotein cholesterol (continuous, mmol/l), low-density lipoprotein cholesterol (continuous, mmol/l), triglycerides (continuous, mmol/l) and blood glucose (continuous, mmol/l). HR, hazard ratio; CI, confidence interval; Ref, reference; y/n, yes/no; VTE, venous thromboembolism.

*Each individual component was modeled as binary variable: met or unmet the healthy criterion. All the five individual components were included in the model simultaneously.

Cox proportional hazards regression analysis of the fully adjusted Model 3 revealed that healthy sleep patterns (5 scores) reduced the risk of PE and DVT by 17.4% and 19.7%, respectively (P < 0.001). Furthermore, healthy sleep behaviors, such as getting 7–8 hours of sleep each day, early chronotype, experiencing infrequent insomnia and daytime sleepiness, were demonstrated to be significantly protective in preventing PE and DVT (P < 0.05) (S1 and S2 Tables).

Sensitivity analyses that omitted participants with missing data on variables (S3 Table) or those with less than 2 years of follow-up (S4 Table) yielded generally robust findings.

### Impact of PRS on the association between sleep behaviors and VTE

**Fig 2:**
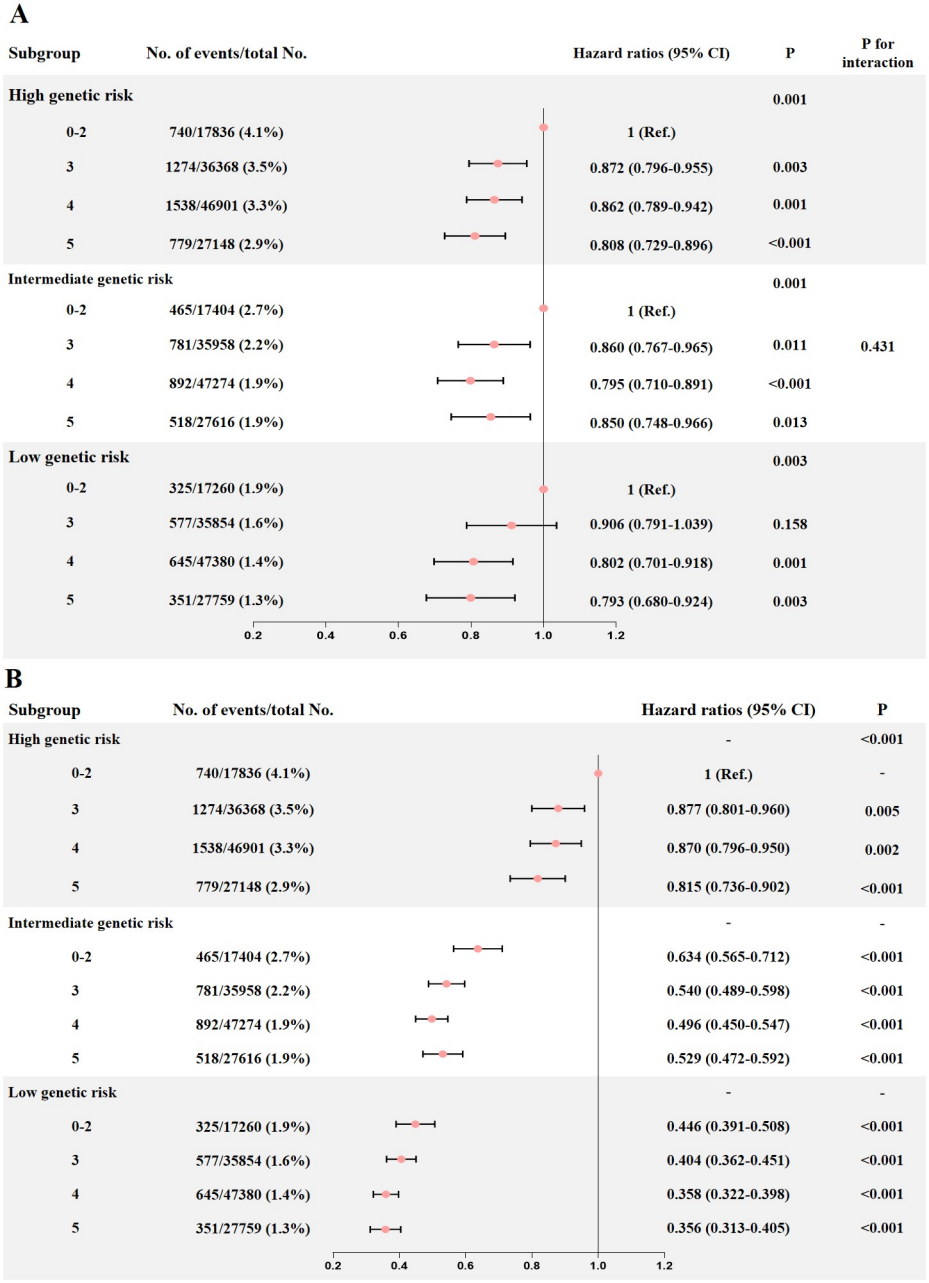
The association of healthy sleep score with VTE in different genetic risk subgroups (A), and the combined association of healthy sleep score and PRS with VTE risk (B).

Participants with a higher genetic risk had a significantly higher incidence of VTE (low vs. intermediate vs. high, 1.5% vs. 2.1% vs. 3.4%, respectively, P<0.001). In the multivariate model (Fig 2), the link between healthy sleep score and VTE proved to be significant (P<0.05) and consistent across individuals in the low, intermediate, and high genetic risk subgroups (P for interaction = 0.431). Comparable correlations were discovered for PE (P for interaction = 0.243) and DVT (P for interaction = 0.627) (S1A and S2A Figs).

We further tested the combined correlation of healthy sleep score and PRS with the incidence of VTE. Participants with a low genetic risk and a good sleep pattern (5 scores) risked least (HR = 0.356, 95% CI: 0.313–0.405, P<0.001) compared to those with a high genetic risk and a poor sleep pattern (0–2 points), who had the greatest risk of VTE (Fig 2B). In a comparable manner, the risk of PE and DVT was decreased by 69.5% and 60.5% (P<0.001), respectively, when low genetic risk and good sleep pattern were combined (S1B and S2B Figs).

## Discussion

Our secondary analysis of a large-scale prospectively gathered registry revealed a robust inverse correlation between healthy sleep patterns and the likelihood of developing VTE. This finding aligns with earlier research that explored the connections between various sleep practices and the risk of cardiovascular disease [23, 29–31]. In addition to conventional risk variables such as demographic, genetic, behavioral, and anthropometric factors [5], individuals with a healthy sleep pattern proved to have 20% lower risk of VTE, including PE and DVT, compared to those with a poor sleep pattern (healthy sleep score = 0–2). A healthy sleep patterns may effectively prevent VTE in the overall population.

Alongside conventional risk variables such as demographic, genetic, behavioral, and anthropometric factors, previous studies have demonstrated that five individual sleep behaviors—early chronotype [9, 10], 7–8 h/day of sleep [7, 8], no snoring [14], infrequent insomnia [11–13], and infrequent daytime sleepiness [15]—are independently linked to a diminished risk of cardiovascular disease (stroke and coronary heart disease, for instance). These findings align with our study on VTE. Furthermore, the correlation between snoring and the occurrence of VTE became insignificant when additional adjustments were made for body mass index. This finding implies that obesity, rather than snoring, is one of the independent risk factors for VTE. Similar findings were obtained from the examination of PE and DVT patients.

We conducted a thorough analysis for the first time to uncover the combined impact of sleep behaviors and PRS on the risk of VTE. Our findings indicate that as genetic susceptibility to VTE (PE and DVT) increases, the protective effect of a healthy sleep score on the risk of VTE remains consistent. A healthy sleep pattern might partly mitigate the occurrence of VTE resulting from a high hereditary vulnerability. In contrast, if a person does not have a healthy sleep pattern, they may lose the innate protection that comes with low genetic risk. Hence, maintaining a healthy sleep pattern is crucial for the primary prevention of VTE and other related complications, irrespective of an individual’s genetic predisposition.

Various mechanisms might contribute to the link between a good sleep pattern and lower VTE risk. By means of multiple mechanisms, unhealthy sleep practices may increase the risk of VTE, either individually or in concert. A relatively later chronotype is linked to several factors that may contribute to VTE, such as mood disorders, [32] smoking habits (33), unhealthy diets [34, 35], and low physical activity [36]. Research has shown a clear association between insufficient sleep duration and many risk factors for VTE, such as autonomic nervous system disorders [16], endothelial dysfunction [37], and metabolic disruption [38]. In addition, an extended period of sleep is linked to elevated levels of markers of systemic inflammation, which may contribute to the development of VTE [39]. Similarly, insomnia is thought to be linked to an elevated heart rate [40] and systemic inflammation [41, 42], which are positively associated with the incidence of VTE [43, 44]. These potential mechanisms are worthy of further research.

The importance of taking sleep practices into account in the prevention of VTE is emphasized by our research. This approach is beneficial for identifying persons at high risk and for facilitating health management. Utilizing a simple scoring algorithm offers a more accessible approach to interpreting epidemiological results and implementing them in real-world scenarios, hence increasing their acceptability among the general populace. Nevertheless, more clinical studies targeting sleep practice interventions will be highly necessary to assess the existence of a cause-and-effect link, given that this research is purely observational and that all the results should be regarded cautiously.

## Limitations

First, the observed connections between sleep patterns and the occurrence of VTE should not be regarded as causative relationships, given the observational nature of this study. Second, this research used a sample consisting of individuals of Caucasian descent from the UK Biobank, thus potentially limiting the generalizability of the findings to groups outside this specific group. Nevertheless, the study’s internal validity will not be undermined. Third, since the sleep habit data included in our study came from a self-report questionnaire, there may be recall bias and misclassification of exposures, which ultimately leads to an underestimation of the impact of the observed relationships. Fourth, it should be noted that sleep behaviors were only recorded at baseline, therefore failing to account for any changes in sleep patterns that may have occurred throughout the course of the evaluation. Fifth, the UK Biobank’s lack of outpatient VTE records, constituting around 15% of VTE cases, and an intra-hospital diagnostic omission rate of about 10% result in a relatively low VTE incidence rate in this study. This limitation may significantly influence the discussion regarding the association between VTE and sleep. However, contemporary D-dimer tests have up to 98% sensitivity for VTE, greatly improving the diagnosis rate of VTE. Finally, the presence of unmeasured or unexplained residual confounding factors is inevitable.

## Conclusions

We identified that healthy sleep patterns, comprising early chronotype, sleeping 7–8 hours each day, infrequent insomnia and daytime sleepiness, are significantly linked with VTE (including PE and DVT) based on data from the UK Biobank study. Our results indicate that a healthy sleep pattern is correlated with a lower risk of developing VTE, irrespective of the individual’s genetic risk. This study highlights the significance of early-stage VTE prevention via the maintenance of a healthy sleep pattern.

## Supporting information

S1 Fig Association of healthy sleep score with PE in subgroups with different genetic risk factors (A), and the joint association of healthy sleep score and PRS with the risk of PE (B). (TIF)

S2 Fig Association of healthy sleep score with DVT in subgroups with different genetic risk factors (A), and the joint association of healthy sleep score and PRS with the risk of DVT (B). (TIF)

S1 Table Association of healthy sleep quality with the risk of PE among 384758 UK Biobank participants. (DOCX)

S2 Table Association of healthy sleep quality with the risk of DVT among 384758 UK Biobank participants. (DOCX)

S3 Table Association of the healthy sleep score with risk of VTE among individuals without missing values. (DOCX)

S4 Table Association of the healthy sleep score with risk of VTE in the individuals with more two years of follow-up. (DOCX)
