# Supplementary figures and images for: Sleep patterns, genetic susceptibility, and venous thromboembolism: A prospective study of 384,758 UK Biobank participants
Source: PLoS One. 2024 Sep 6;19(9):e0309870. doi: 10.1371/journal.pone.0309870 (PMC11379228; doi:10.1371/journal.pone.0309870)

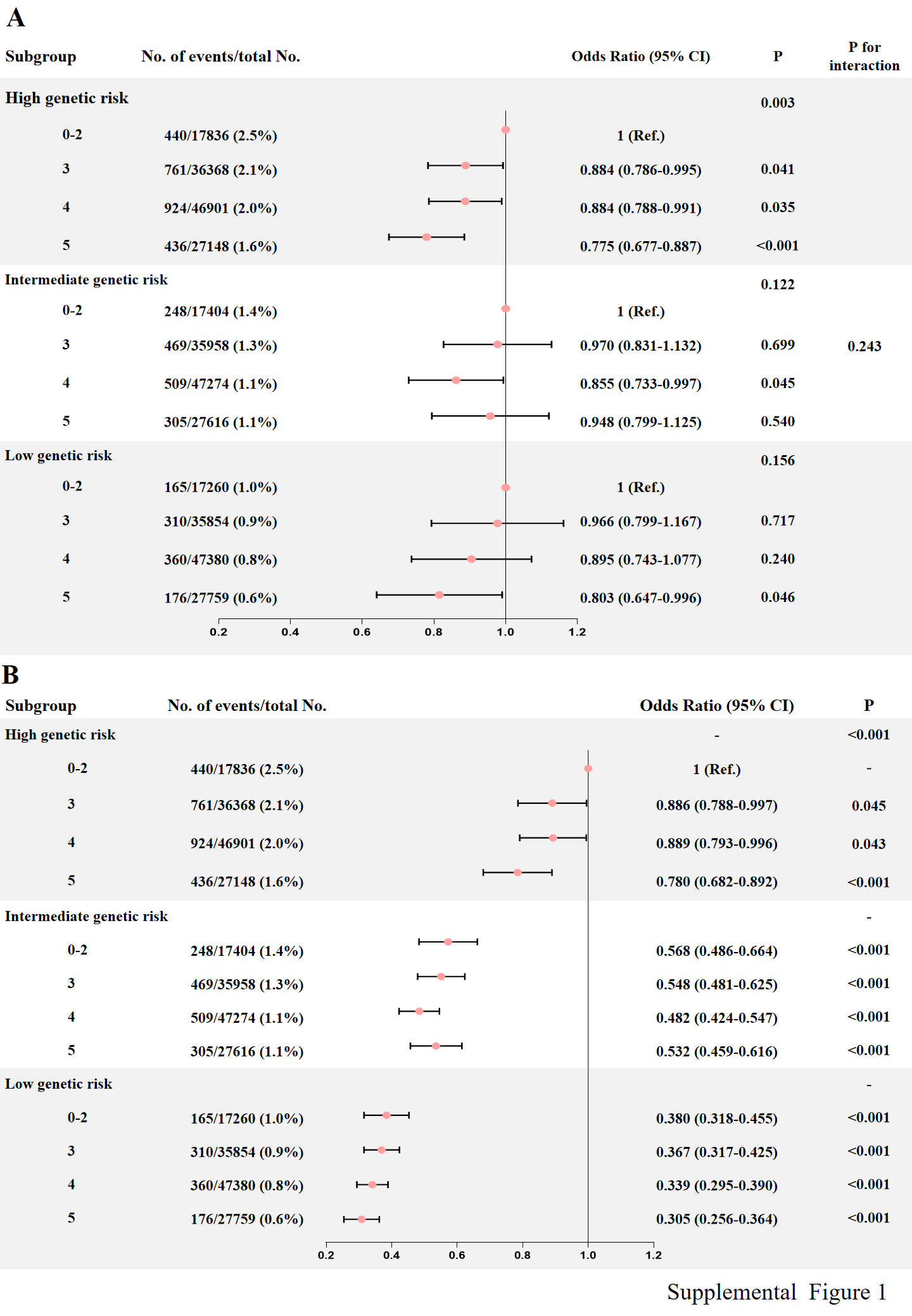

Supplement: S1 Fig — (TIF) [file pone.0309870.s001.tif]

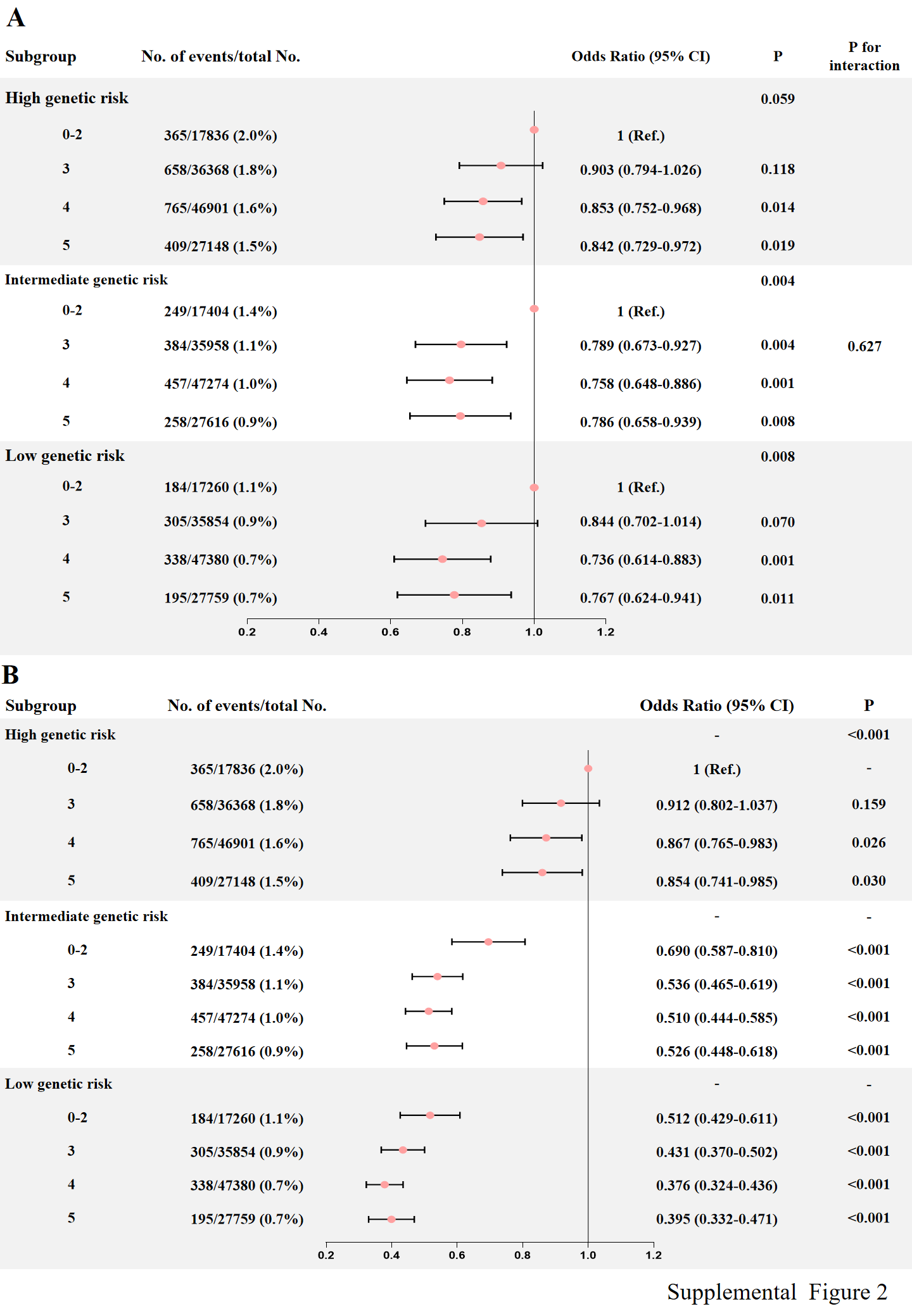

Supplement: S2 Fig — (TIF) [file pone.0309870.s002.tif]
