# Supplementary material for: Sleep patterns, genetic susceptibility, and venous thromboembolism: A prospective study of 384,758 UK Biobank participants
Source: PLoS One. 2024 Sep 6;19(9):e0309870. doi: 10.1371/journal.pone.0309870 (PMC11379228; doi:10.1371/journal.pone.0309870)
Supplement: S1 Table — (DOCX) [file pone.0309870.s003.docx]

**S1 Table.** Association of the healthy sleep score with risk of PE among 384758 UK Biobank participants.

| Sleep behaviors | | No. of events/total No.  (n, %) | Model 1 | | Model 2 | | Model 3 | |
| --- | --- | --- | --- | --- | --- | --- | --- | --- |
|  |  |  | HR (95% CI) | P | HR (95% CI) | P | HR (95% CI) | P |
| **Healthy sleep score** | |  |  | <0.001 |  | <0.001 |  | <0.001 |
|  | 0-2 | 853/52500 (1.6%) | Reference | - | Reference | - | Reference | - |
|  | 3 | 1540/108180 (1.4%) | 0.867 (0.798 -0.943) | 0.001 | 0.877 (0.806-0.953) | 0.002 | 0.926 (0.852-1.007) | 0.074 |
|  | 4 | 1793/141555 (1.3%) | 0.768 (0.708-0.833) | <0.001 | 0.800 (0.737-0.868) | <0.001 | 0.874 (0.805-0.949) | 0.001 |
|  | 5 | 917/82523 (1.1%) | 0.670 (0.611-0.736) | <0.001 | 0.724 (0.660-0.795) | <0.001 | 0.826 (0.751-0.908) | <0.001 |
|  | Per 1 point |  | 0.881 (0.858-0.904) | <0.001 | 0.903 (0.879-0.927) | <0.001 | 0.935 (0.913-0.958) | <0.001 |
| **Individual component*** | |  |  |  |  |  |  |  |
| Chronotype | |  |  |  |  |  |  |  |
|  | Late chronotype | 1942/143370(1.4%) | Reference |  | Reference |  | Reference |  |
|  | Early chronotype | 3161/241388 (1.3%) | 0.970 (0.916-1.026) | 0.286 | 0.889 (0.840-0.941) | **<0.001** | 0.917 (0.866-0.971) | **0.003** |
| Sleep duration | |  |  |  |  |  |  |  |
|  | <7h/d or 8h/d< | 1814/121259 (1.5%) | Reference |  | Reference |  | Reference |  |
|  | 7–8 h/d | 3289/263499 (1.2%) | 0.853 (0.804-0.905) | **<0.001** | 0.898 (0.846-0.953) | **<0.001** | 0.926 (0.873-0.983) | **0.011** |
| Frequent insomnia | |  |  |  |  |  |  |  |
|  | Yes | 1580/107723 (1.5%) | Reference |  | Reference |  | Reference |  |
|  | No | 3523/277035 (1.3%) | 0.903 (0.850-0.961) | **0.001** | 0.944 (0.888-1.004) | 0.068 | 0.969 (0.911-1.031) | 0.325 |
| Snoring | |  |  |  |  |  |  |  |
|  | Yes | 2074/142768 (1.5%) | Reference |  | Reference |  | Reference |  |
|  | No | 3029/241990 (1.3%) | 0.866 (0.819-0.916) | **<0.001** | 0.923 (0.872-0.977) | **0.006** | 0.996 (0.940-1.055) | 0.890 |
| Frequent daytime sleepiness | |  |  |  |  |  |  |  |
|  | Yes | 214/10116 (2.1%) | Reference |  | Reference |  | Reference |  |
|  | No | 4889/374641 (1.3%) | 0.644 (0.561-0.739) | **<0.001** | 0.723 (0.630-0.830) | **<0.001** | 0.761 (0.622-0.873) | **<0.001** |

Model 1 is univariable Cox regression analysis.

Model 2 is adjusted by age (continuous, years), sex (male, female), education (College or University degree, A levels/AS levels or equivalent, O levels/GCSEs or equivalent, Other (e.g.NVO,nursing,missing)), annual household income (<£18 000, £18 000 to £52 000, >£52 000).

Model 3 is adjusted by model 2 plus body mass index (continuous, kg/m2), physical activity (continuous, MET-hours/week) , smoking (never, former, current), drinking (never, former, current), hypertension (y/n), diabetes (y/n), cancer (y/n), cardiovascular disease (y/n), total cholesterol (continuous, mmol/l), high density lipoprotein cholesterol(continuous, mmol/l), low density lipoprotein cholesterol (continuous, mmol/l), triglycerides (continuous, mmol/l) and blood glucose (continuous, mmol/l). HR indicates hazard ratio; CI, confidence interval; Ref, reference; and y/n, yes/no; PE, pulmonary embolism.

*Each individual component was modeled as binary variable: met or unmet the healthy criterion. All the five individual components were included in the model simultaneously.
