# Supplementary material for: Sleep patterns, genetic susceptibility, and venous thromboembolism: A prospective study of 384,758 UK Biobank participants
Source: PLoS One. 2024 Sep 6;19(9):e0309870. doi: 10.1371/journal.pone.0309870 (PMC11379228; doi:10.1371/journal.pone.0309870)
Supplement: S2 Table — (DOCX) [file pone.0309870.s004.docx]

**S2 Table.** Association of the healthy sleep score with risk of DVT among 384758 UK Biobank participants.

| Sleep behaviors | | No. of events/total No.  (n, %) | Model 1 | | Model 2 | | Model 3 | |
| --- | --- | --- | --- | --- | --- | --- | --- | --- |
|  |  |  | HR (95% CI) | P | HR (95% CI) | P | HR (95% CI) | P |
| **Healthy sleep score** | |  |  | <0.001 |  | <0.001 |  | <0.001 |
|  | 0-2 | 798/52500 (1.5%) | Reference | - | Reference | - | Reference | - |
|  | 3 | 1347/108180 (1.2%) | 0.812 (0.744 -0.886) | <0.001 | 0.820 (0.752-0.895) | <0.001 | 0.855 (0.783-0.933) | <0.001 |
|  | 4 | 1560/141555 (1.1%) | 0.715 (0.656-0.778) | <0.001 | 0.744 (0.683-0.810) | <0.001 | 0.794 (0.728-0.865) | <0.001 |
|  | 5 | 862/82523 (1.0%) | 0.675 (0.613-0.743) | <0.001 | 0.732 (0.665-0.807) | <0.001 | 0.803 (0.728-0.886) | <0.001 |
|  | Per 1 point |  | 0.884 (0.860-0.909) | <0.001 | 0.907 (0.882-0.933) | <0.001 | 0.933 (0.907-0.960) | <0.001 |
| **Individual component*** | |  |  |  |  |  |  |  |
| Chronotype | |  |  |  |  |  |  |  |
|  | Late chronotype | 1751/143370 (1.2%) | Reference |  | Reference |  | Reference |  |
|  | Early chronotype | 2816/241388 (1.2%) | 0.957 (0.902-1.016) | 0.150 | 0.889 (0.838-0.944) | **<0.001** | 0.907 (0.854-0.963) | **0.001** |
| Sleep duration | |  |  |  |  |  |  |  |
|  | <7h/d or 8h/d< | 1622/121259 (1.3%) | Reference |  | Reference |  | Reference |  |
|  | 7–8 h/d | 2945/263499 (1.1%) | 0.855 (0.804-0.911) | **<0.001** | 0.898 (0.843-0.956) | **0.001** | 0.921 (0.865-0.981) | **0.010** |
| Frequent insomnia | |  |  |  |  |  |  |  |
|  | Yes | 1427/107723 (1.3%) | Reference |  | Reference |  | Reference |  |
|  | No | 3140/277035 (1.1%) | 0.888 (0.833-0.948) | **<0.001** | 0.914 (0.857-0.975) | **0.007** | 0.934 (0.875-0.997) | **0.040** |
| Snoring | |  |  |  |  |  |  |  |
|  | Yes | 1836/142768 (1.3%) | Reference |  | Reference |  | Reference |  |
|  | No | 2731/241990 (1.1%) | 0.880 (0.830-0.934) | **<0.001** | 0.954 (0.898-1.013) | 0.125 | 0.999 (0.940-1.063) | 0.984 |
| Frequent daytime sleepiness | |  |  |  |  |  |  |  |
|  | Yes | 172/10116 (1.7%) | Reference |  | Reference |  | Reference |  |
|  | No | 4395/374641 (1.2%) | 0.722 (0.619-0.841) | **<0.001** | 0.801 (0.687-0.933) | **0.005** | 0.842 (0.722-0.982) | **0.029** |

Model 1 is univariable Cox regression analysis.

Model 2 is adjusted by age (continuous, years), sex (male, female), education (College or University degree, A levels/AS levels or equivalent, O levels/GCSEs or equivalent, Other (e.g.NVO,nursing,missing)), annual household income (<£18 000, £18 000 to £52 000, >£52 000).

Model 3 is adjusted by model 2 plus body mass index (continuous, kg/m2), physical activity (continuous, MET-hours/week) , smoking (never, former, current), drinking (never, former, current), hypertension (y/n), diabetes (y/n), cancer (y/n), cardiovascular disease (y/n), total cholesterol (continuous, mmol/l), high density lipoprotein cholesterol(continuous, mmol/l), low density lipoprotein cholesterol (continuous, mmol/l), triglycerides (continuous, mmol/l) and blood glucose (continuous, mmol/l). HR indicates hazard ratio; CI, confidence interval; Ref, reference; and y/n, yes/no; DVT, deep vein thrombosis.

*Each individual component was modeled as binary variable: met or unmet the healthy criterion. All the five individual components were included in the model simultaneously.
