# Supplementary material for: Sleep patterns, genetic susceptibility, and venous thromboembolism: A prospective study of 384,758 UK Biobank participants
Source: PLoS One. 2024 Sep 6;19(9):e0309870. doi: 10.1371/journal.pone.0309870 (PMC11379228; doi:10.1371/journal.pone.0309870)
Supplement: S3 Table — (DOCX) [file pone.0309870.s005.docx]

**S3 Table.** Association of the healthy sleep score with risk of VTE among individuals without missing values

| Sleep behaviors | | No. of events/total No.  (n, %) | Model 1 | | Model 2 | | Model 3 | |
| --- | --- | --- | --- | --- | --- | --- | --- | --- |
|  |  |  | HR (95% CI) | P | HR (95% CI) | P | HR (95% CI) | P |
| **Healthy sleep score** | |  |  | <0.001 |  | <0.001 |  | <0.001 |
|  | 0-2 | 1086/38638 (2.8%) | Reference | - | Reference | - | Reference | - |
|  | 3 | 1971/80255 (2.5%) | 0.866 (0.804 -0.933) | <0.001 | 0.877 (0.815-0.945) | 0.001 | 0.919 (0.853-0.990) | 0.026 |
|  | 4 | 2263/105055 (2.2%) | 0.756 (0.703-0.813) | <0.001 | 0.791 (0.736-0.850) | <0.001 | 0.851 (0.791-0.916) | <0.001 |
|  | 5 | 1175/60765 (1.9%) | 0.675 (0.621-0.733) | <0.001 | 0.736 (0.678-0.800) | <0.001 | 0.820 (0.754-0.892) | <0.001 |
|  | Per 1 point |  | 0.881 (0.860-0.901) | <0.001 | 0.905 (0.884-0.926) | <0.001 | 0.935 (0.913-0.958) | <0.001 |
| **Individual component*** | |  |  |  |  |  |  |  |
| Chronotype | |  |  |  |  |  |  |  |
|  | Late chronotype | 2487/105647 (2.4%) | Reference |  | Reference |  | Reference |  |
|  | Early chronotype | 4008/179066 (2.2%) | 0.953 (0.906-1.002) | 0.058 | 0.880 (0.837-0.926) | **<0.001** | 0.902 (0.857-0.949) | **<0.001** |
| Sleep duration | |  |  |  |  |  |  |  |
|  | <7h/d or 8h/d< | 2287/89183 (2.6%) | Reference |  | Reference |  | Reference |  |
|  | 7–8 h/d | 4208/195530 (2.2%) | 0.856 (0.812-0.902) | **<0.001** | 0.902 (0.855-0.950) | **<0.001** | 0.925 (0.877-0.975) | **0.004** |
| Frequent insomnia | |  |  |  |  |  |  |  |
|  | Yes | 1994/79275 (2.5%) | Reference |  | Reference |  | Reference |  |
|  | No | 4501/205438 (2.2%) | 0.904 (0.856-0.955) | **<0.001** | 0.939 (0.889-0.992) | **0.024** | 0.962 (0.910-1.016) | **0.163** |
| Snoring | |  |  |  |  |  |  |  |
|  | Yes | 2686/107147 (2.5%) | Reference |  | Reference |  | Reference |  |
|  | No | 3809/177566 (2.1%) | 0.859 (0.818-0.903) | **<0.001** | 0.925 (0.879-0.973) | **<0.001** | 0.983 (0.934-1.035) | 0.520 |
| Frequent daytime sleepiness | |  |  |  |  |  |  |  |
|  | Yes | 251/7441 (3.4%) | Reference |  | Reference |  | Reference |  |
|  | No | 6244/277272 (2.3%) | 0.689 (0.614-0.792) | **<0.001** | 0.784 (0.690-0.890) | **<0.001** | 0.821 (0.722-0.932) | **0.002** |

Model 1 is univariable Cox regression analysis.

Model 2 is adjusted by age (continuous, years), sex (male, female), education (College or University degree, A levels/AS levels or equivalent, O levels/GCSEs or equivalent, Other (e.g.NVO,nursing,missing)), annual household income (<£18 000, £18 000 to £52 000, >£52 000).

Model 3 is adjusted by Model 2 plus body mass index (continuous, kg/m2), physical activity (continuous, MET-hours/week) , smoking (never, former, current), drinking (never, former, current), hypertension (y/n), diabetes (y/n), cancer (y/n), cardiovascular disease (y/n), total cholesterol (continuous, mmol/l), high density lipoprotein cholesterol(continuous, mmol/l), low density lipoprotein cholesterol (continuous, mmol/l), triglycerides (continuous, mmol/l) and blood glucose (continuous, mmol/l). HR indicates hazard ratio; CI, confidence interval; Ref, reference; and y/n, yes/no; VTE, venous thromboembolism.

*Each individual component was modeled as binary variable: met or unmet the healthy criterion. All the five individual components were included in the model simultaneously.
