# Supplementary material for: Sleep patterns, genetic susceptibility, and venous thromboembolism: A prospective study of 384,758 UK Biobank participants
Source: PLoS One. 2024 Sep 6;19(9):e0309870. doi: 10.1371/journal.pone.0309870 (PMC11379228; doi:10.1371/journal.pone.0309870)
Supplement: S4 Table — (DOCX) [file pone.0309870.s006.docx]

**S4 Table.** Association of the healthy sleep score with risk of VTE in the individuals with more two years of follow-up

| Sleep behaviors | | No. of events/total No.  (n, %) | Model 1 | | Model 2 | | Model 3 | |
| --- | --- | --- | --- | --- | --- | --- | --- | --- |
|  |  |  | HR (95% CI) | P | HR (95% CI) | P | HR (95% CI) | P |
| **Healthy sleep score** | |  |  | <0.001 |  | <0.001 |  | <0.001 |
|  | 0-2 | 1367/52009 (2.6%) | Reference | - | Reference | - | Reference | - |
|  | 3 | 1971/107443 (2.2%) | 0.839 (0.785-0.897) | <0.001 | 0.848 (0.794-0.906) | <0.001 | 0.889 (0.832-0.950) | 0.001 |
|  | 4 | 2263/140698 (2.0%) | 0.742 (0.696-0.792) | <0.001 | 0.773 (0.725-0.825) | <0.001 | 0.834 (0.781-0.890) | <0.001 |
|  | 5 | 1479/82079 (1.8%) | 0.674 (0.626-0.725) | <0.001 | 0.728 (0.676-0.784) | <0.001 | 0.812 (0.754-0.875) | <0.001 |
|  | Per 1 point |  | 0.881 (0.863-0.900) | <0.001 | 0.903 (0.884-0.922) | <0.001 | 0.934 (0.914-0.954) | <0.001 |
| **Individual component*** | |  |  |  |  |  |  |  |
| Chronotype | |  |  |  |  |  |  |  |
|  | Late chronotype | 3045/142372 (2.1%) | Reference |  | Reference |  | Reference |  |
|  | Early chronotype | 4972/239857 (2.1%) | 0.972 (0.930-1.017) | 0.224 | 0.895 (0.855-0.936) | **<0.001** | 0.917 (0.876-0.960) | **<0.001** |
| Sleep duration | |  |  |  |  |  |  |  |
|  | <7h/d or 8h/d< | 2834/120228 (2.4%) | Reference |  | Reference |  | Reference |  |
|  | 7–8 h/d | 5183/262001 (2.0%) | 0.861 (0.822-0.903) | **<0.001** | 0.905 (0.863-0.949) | **<0.001** | 0.931 (0.887-0.967) | **0.003** |
| Frequent insomnia | |  |  |  |  |  |  |  |
|  | Yes | 2500/106877 (2.3%) | Reference |  | Reference |  | Reference |  |
|  | No | 5517/275352 (2.0%) | 0.889 (0.847-0.934) | **<0.001** | 0.927 (0.883-0.974) | **0.002** | 0.949 (0.903-0.997) | **0.036** |
| Snoring | |  |  |  |  |  |  |  |
|  | Yes | 3271/141863 (2.3%) | Reference |  | Reference |  | Reference |  |
|  | No | 4746/240366 (2.0%) | 0.859 (0.822-0.899) | **<0.001** | 0.918 (0.877-0.961) | **<0.001** | 0.975 (0.931-1.021) | 0.288 |
| Frequent daytime sleepiness | |  |  |  |  |  |  |  |
|  | Yes | 320/9993 (3.2%) | Reference |  | Reference |  | Reference |  |
|  | No | 7697/372235 (2.1%) | 0.678 (0.606-0.759) | **<0.001** | 0.756 (0.675-0.846) | **<0.001** | 0.794 (0.709-0.889) | **<0.001** |

Model 1 is univariable Cox regression analysis.

Model 2 is adjusted by age (continuous, years), sex (male, female), education (College or University degree, A levels/AS levels or equivalent, O levels/GCSEs or equivalent, Other (e.g.NVO,nursing,missing)), annual household income (<£18 000, £18 000 to £52 000, >£52 000).

Model 3 is adjusted by Model 2 plus body mass index (continuous, kg/m2), physical activity (continuous, MET-hours/week) , smoking (never, former, current), drinking (never, former, current), hypertension (y/n), diabetes (y/n), cancer (y/n), cardiovascular disease (y/n), total cholesterol (continuous, mmol/l), high density lipoprotein cholesterol(continuous, mmol/l), low density lipoprotein cholesterol (continuous, mmol/l), triglycerides (continuous, mmol/l) and blood glucose (continuous, mmol/l). HR indicates hazard ratio; CI, confidence interval; Ref, reference; and y/n, yes/no; VTE, venous thromboembolism.

*Each individual component was modeled as binary variable: met or unmet the healthy criterion. All the five individual components were included in the model simultaneously.
